# Intestinal Parasitic Infection and Associated Risk Factors among HIV-Infected Patients Seeking Healthcare in a Rural Hospital in Ghana

**DOI:** 10.1155/2022/5652637

**Published:** 2022-08-22

**Authors:** John Gameli Deku, Kwesi Amoah Botchway, Silas Kinanyok, Charles Kwame Gedzeah, Richard Vikpebah Duneeh, Kwabena Obeng Duedu

**Affiliations:** ^1^Department of Medical Laboratory Sciences, School of Allied Health Sciences, University of Health and Allied Sciences, Ho, Ghana; ^2^Laboratory Department, St. Mary Theresa Hospital, Dodi Papase, Ghana; ^3^Department of Biomedical Science, School of Basic and Biomedical Sciences, University of Health and Allied Sciences, Ho, Ghana

## Abstract

**Background:**

Parasitic infections among human immunodeficiency virus (HIV)-infected patients are common in various regions and populations across the world and have since remained a persistent public health challenge. Sub-Saharan Africa harbors the greatest burden of the infections due to sociodemographic and behavioral factors. However, the prevalence of gastrointestinal parasitic infections among HIV-infected persons has been poorly investigated in Ghana.

**Aim:**

This study sought to determine the prevalence of gastrointestinal parasitic infections and associated factors in HIV-infected individuals attending the antiretroviral therapy (ART) clinic in St. Mary Theresa Hospital, Dodi Papase.

**Methods:**

A cross-sectional study was conducted from June 2021 to September 2021 among three hundred and thirty-five HIV-infected individuals in the study area. Sociodemographic and behavioral factors were collected with the aid of a close-ended structured questionnaire. Furthermore, stool samples were collected from each participant and examined for the presence of intestinal parasites by microscopy using direct wet mount, formol-ether concentration, and modified Ziehl–Neelsen (Zn) techniques. Data obtained were analyzed using Statistical Package for Social Sciences (SPSS) version 22.0 and Graphpad Prism version 8.

**Results:**

The prevalence of gastrointestinal parasitic infections was 5.97%. Species-specific prevalence was found to be 2.99% for *Giardia lamblia*, 1.19% for *Ascaris lumbricoides,* and 0.90% each for *Entamoeba histolytica/dispar* and *Trichuris trichiura*. There was a significant association between participants' educational level and intestinal parasitic infection. In addition, gastrointestinal parasitic infections were not found to be associated with age. Unemployed participants, those with a lower frequency of deworming, and those who do not use water closet toilet facilities were at a higher risk of getting infected.

**Conclusion:**

The lower infection rate recorded in this study suggests that public health interventions put in place are yielding significant results. Even though the prevalence is low, routine screening of all HIV-infected patients for parasitic infection is recommended to ensure timely, effective treatment and comprehensive care.

## 1. Introduction

Human immunodeficiency virus (HIV) infections have proven to be one of the most complex and devastating infectious diseases ever discovered in the history of humans due to its complicated pathophysiology and association with other infectious diseases [1]. Intestinal parasitic infections, on the other hand, remain one of the world's most widespread of all chronic human infections and essentially cause high morbidity and mortality across low- and middle-income countries [2, 3].

Parasitic infections among HIV-infected patients are common in various regions and populations across the world and have since remained a persistent public health challenge [4, 5]. Intestinal parasitic infection rates from previous studies ranged from 15% to 80% among people living with HIV in developing nations [6, 7]. Particularly, the rate of parasitic infection among HIV/AIDS patients is markedly high in sub-Saharan Africa, where the majority of HIV/AIDS infection cases are concentrated [8, 9].

Deterioration of the immune system due to HIV infection is what predisposes patients to numerous opportunistic infections, including gastrointestinal parasitic infections [1]. Most intestinal parasites, otherwise considered nonpathogenic or present with transient pathogenic potential in immunocompetent individuals, turn to become more aggressive and cause devastating illnesses in HIV/AIDS patients due to their compromised immunity [3, 10].

Several studies have shown that intestinal parasitic infections are the leading cause of morbidity and mortality in patients infected with HIV/AIDS [1, 4, 11]. Approximately, 80% of AIDS patients' deaths are attributable to coinfection with other infectious agents, including intestinal parasites [3]. The introduction of antiretroviral therapy has proven significant effectiveness in reducing the burden of gastrointestinal infections in HIV-positive patients [5]; nonetheless, most people living with HIV infection still suffer from gastrointestinal parasitic infections as the prevalence rates continue to remain significantly high [6, 7]. Lack of sanitary facilities and lack of health education are major reasons reported to facilitate the transmission of intestinal parasites, especially among HIV patients [1, 12].

The ecology of intestinal parasitism among HIV patients in Ghana is quite wide, but there is meagre public knowledge about the prevalence of HIV and parasitic coinfections and the associated predisposing factors. Hence, there is a need for an up-to-date surveillance of intestinal parasitic infections among HIV-positive patients to provide adequate information for effective management and prognosis to reduce the rate of morbidity and mortality. In this light, the current study was conducted to determine the intestinal parasitic prevalence and associated risk factors in the HIV-infected population seeking healthcare at St. Mary Theresa Hospital in Dodi Papase located in the Kadjebi District of Ghana.

## 2. Materials and Methods

### 2.1. Study Design and Site

**Figure 1:**
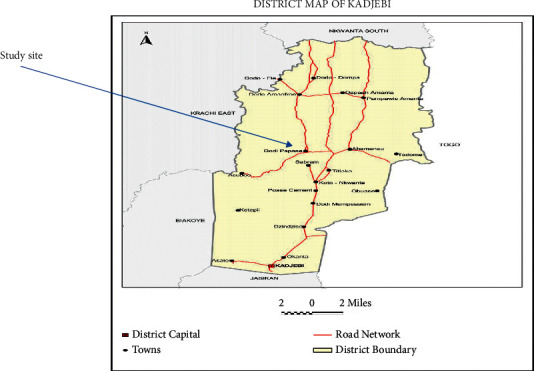
Map of Kadjebi District showing the study site [13].

This cross-sectional study using convenient sampling was conducted at the antiretroviral therapy (ART) clinic and Laboratory Department of the St. Mary Theresa Hospital from June 2021 to September 2021. The hospital is in Dodi Papase, a major town in the Kadjebi District of the Oti Region of Ghana (Figure 1). The town lies within latitude 5° 33′ 35.5” (5.5599°) North and longitude 0° 12′ 45.7” (0.2127°) North and shares boundaries with Biakoye, Jasikan, and Nkwanta South districts to the east, south, and north, respectively. St. Mary Theresa Hospital serves as the main referral hospital in the area and admits patients across the entire district and the neighboring districts.

### 2.2. Study Population

The study population constituted the subjects who were HIV positive and were visiting the ART Clinic at St Mary Theresa Hospital, Dodi Papase, for routine check-up, collection of medications, or other medical complaints. Patients who willingly gave informed consent and volunteered to have their stool samples examined were thus recruited into the study.

### 2.3. Data Collection

Data were collected from study participants by use of a structured questionnaire which contained information on the demographic characteristics, socioeconomic parameters, and the type of toilet facility used among others. Questionnaire used in the study can be found in Supplementary Table 1. English, Ewe, and Twi were the main languages employed to communicate the interview and questionnaire. However, for study participants who could not speak or understand any of these languages, the necessary assistance was sought from an interpreter.

### 2.4. Sample Collection and Laboratory Analysis

Fresh stool samples were collected using a wide-mouth sterile plastic container from each participant and labeled with the participant's details for laboratory examination. Wet mount preparation, formol-ether concentration technique, and modified Ziehl–Neelsen method of stool preparation were carried out to detect the presence of intestinal parasites, eggs or cysts, and oocysts [14].

### 2.5. Laboratory Techniques

#### 2.5.1. Wet Mount Method

With the help of an applicator, a piece of stool was picked and emulsified with a drop of normal saline on a glass slide. A cover glass was then gently placed on it to avoid air bubbles, and the preparation was examined under the microscope.

#### 2.5.2. Formol-Ether Concentration Method

With a small stick, an estimated 1 g (pea-size) of stool was emulsified in 4 ml of 10% formol water contained in a screw-cap tube and topped with 4 ml of 10% v/v formol water, capped, and mixed by shaking. The collected suspension was transferred into a conical (centrifuge) tube made of strong glass, copolymer, or polypropylene, and 3 ml of diethyl ether (ethyl acetate) was added, stoppered, mixed for 1 minute, and centrifuged at 3000 rpm for 1 minute. With a stick, the layer of faecal debris was loosened from the side of the tube and the tube was inverted to discard the ether, faecal debris, and formol water leaving the sediment at the bottom. The tube is returned to its upright position, and the fluid is allowed to drain from the side to the bottom. The bottom of the tube was tapped to resuspend and mix the sediment. Part of the sediment was then transferred onto a slide, covered with a cover glass, and examined microscopically for the presence of cysts and eggs [15].

#### 2.5.3. Modified Ziehl–Neelsen Method

A smear was prepared from the sediment obtained by the formol-ether concentration technique. After air-drying and fixing with methanol for 3 minutes, it was stained with unheated carbol fuchsin for 15 minutes and 0.5% methylene blue was used to stain for 30 seconds as a counter stain, following 10 seconds of decolorization with 1% acid alcohol.

### 2.6. Statistical Analysis

The information recorded in the questionnaire and results from the laboratory investigations were checked for completeness and accuracy. Entries from the questionnaires were double-entered into a predesigned electronic database using the Fox-Pro interface, verified, and cleaned. The verified and cleaned data set was exported to IBM Statistical Package for the Social Sciences (SPSS Inc., Chicago, USA) version 22.00 and GraphPad Prism version 6.00 for Windows (GraphPad Software, San Diego USA).

Descriptive methods of analysis such as tables and graphs were used to analyze and illustrate the occurrence of the various factors in the study. Categorical data were analyzed using the Chi-square (X) and Fisher's exact *t*-test. The associations between gastrointestinal parasitic infections and possible risk factors were determined using binary logistic regression and described in terms of odds ratio (OR) at 95% confidence interval (CI). Independent risk factors for the occurrence of intestinal parasitic infections were identified with the logistic regression. A *P* value of <0.05 was statistically significant for all statistical comparisons.

### 2.7. Ethical Consideration

Ethical clearance was obtained from the Research Ethics Committee of the University of Health and Allied Sciences, with protocol number UHAS-REC A.10 [57] 20–21. Written permission was also sought from the authorities of the St. Mary Theresa Hospital, Dodi Papase. Written consent was obtained from all participants before the commencement of the study. Confidentiality of participants' information and data obtained from the study was ensured.

## 3. Results

The study recruited a total of 335 participants, of whom 66.57% of them were above youthful age (>35 years) at the time of the study. The analysis also showed that the majority of the participants (75.22%) did not have any form of formal education, with only 0.60% of them having attained tertiary education. Approximately half (50.15%) the number of the participants professed to be married and 54.33% of them also indicated they were actively employed.

Regarding their hygiene and lifestyle practices, the majority (64.18%) of them used pit latrines. The main source of feeding for most of the participants was through homemade dishes (91.34%), while the remaining practiced buying food from outside. About 59.10% of the participants drink sachet water, which serves as their main source of drinking water. The rest drink from either a borehole, river, or a combination of any two or three. Details of these results are presented in Table 1.

The overall prevalence of intestinal parasitic infection among the study participants stood at 5.97% involving four different species of parasites. The specific prevalence of each of the parasitic agents retrieved was 2.99% for *Giardia lamblia*, 1.19% for *Ascaris lumbricoides*, and 0.90% each of *Trichuris trichiura* and *Entamoeba histolytica*/*dispar*.

About 4.78% of the parasitic agents were identified from the wet preparation technique, while 2.09% were recovered from the formol-ether concentration technique. In terms of the stage of the parasitic agents, the percentage of trophozoites and cysts retrieved was 3.28% and 2.09%, all from the wet preparation and formol-ether concentration, respectively. Moreover, the percentage of eggs identified was 1.49% from formol-ether concentration as shown in Table 2.

**Figure 2:**
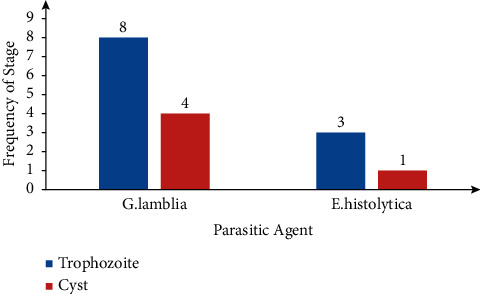
Distribution of stages of protozoan parasitic agents.

**Figure 3:**
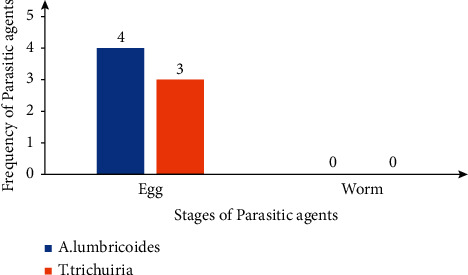
Distribution of stages of helminthic parasitic agents.

For the stages of the parasitic agents, both trophozoites and cysts of *Giardia lamblia* and *Entamoeba histolytica/dispar* were retrieved (Figure 2). However, for *Ascaris lumbricoides* and *Trichuris trichiura*, only eggs were retrieved, as seen in Figure 3.

Logistic regression analysis showed a significant variation with regard to participants' level of education and infection with any parasitic agent. However, regarding analysis of the odds using tertiary education attainment as the reference, there were no statistical significant variations in terms of the participants' risk of infection with parasitic agents and their educational levels. Moreover, there was no statistical significant variation with regard to the participants' marital and employment statuses and their chances of getting parasitic infections. The odds ratios, however, revealed that being single presents twice the risk of getting infected with any of the parasitic agents compared to being married, although not statistically significant (OR: 2 : 1, CI = 0.275 to 15.927, *P* value = 0.475). Furthermore, participants with active employment status were significantly at higher risk of getting infected than those who were unemployed (OR: 6 : 1, CI = 1.082 to37.123, *P* value = 0.041).

For hygiene, lifestyle, and history, the deworming history exhibited a very strong significant variation in the participants' frequencies of deworming and infection with parasitic agents. Using none as the reference, the odds analysis revealed a lower participant risk of getting a parasitic infection for any history of deworming compared to no history, except for 3 months or below who had an odds value of 20 : 1, although not significant (*P* value = 0.097). There were significant variations observed for the type of toilet facility used by the study participants, and the same was observed about their source of feeding and source of drinking water. Nonetheless, for the odds ratio analysis, using the use of a water closet as the reference, the risks of getting infected as one who uses a pit latrine or practices open defecation were (OR = 53 : 1, CI = 1.512 to 1858.251, *P* value = 0.029) and (OR: 12 : 1, CI = 0.390 to 379.756, *P* value = 0.155), as shown in Table 3.

## 4. Discussion

Intestinal parasitic infections among HIV patients remain a major public health burden. Although the introduction of ART has yielded some appreciable results, the rate of morbidity and mortality caused by intestinal parasitic coinfection with HIV remains significantly high. The prevalence of intestinal parasitic infection among the study population in the current study was 5.97% involving the four main species of parasites, *Giardia lamblia, Trichuris trichiura*, *Ascaris lumbricoides*, and *Entamoeba histolytica/dispar*.

The parasitic prevalence in the current study is lower than in previous reports in Ghana [16, 17], parts of Africa [2, 8, 9], and other parts of the world [18, 19]. The lower prevalence in the current study than in the other previous studies may be attributed to the periodic mass screening of food vendors in the study area and the consequential treatment of positive cases among them, an occupation most of the study participants are engaged in, especially as the study was conducted around the same time these screening exercises are conducted. Furthermore, the outcome can be attributed to effective counseling and continuous education on hygienic practices for HIV seropositive individuals by health professionals.

In this current study, *Giardia lamblia* was the predominant parasitic organism recovered from the stool samples. Our finding is in agreement with previous studies conducted in Ghana [16, 17] and other parts of the world [20–22]. *Giardia lamblia* is a medically important gastrointestinal parasite associated with diarrhea. They are especially found in communities without proper sanitation and potable water. Without periodical diagnosis, these people may serve as reservoirs of the organism and pass on the parasite to a susceptible host [23]. Although infection with *Giardia lamblia* and HIV correlated with enteritis or enterocolitis, its incidence does not differ amongst HIV-positive and -negative patient populations [24, 25].

In terms of the demographic characteristics of the participants, there were no significant differences in infection rate with regard to age and marital status, although we recorded a higher proportion of older folks being infected, similar to findings from previous studies [5, 16]. That notwithstanding, our study revealed that participants with lower levels of education or no education at all were significantly infected with intestinal parasites than their counterparts with higher education. This is consistent with the outcome obtained in previous studies conducted in Nigeria [4, 12]. Again, in terms of hygiene practice and history of deworming, participants who did not practice regular deworming were at a higher risk of acquiring intestinal parasitic infections. The source of drinking water was not found to be significantly associated with intestinal parasitic infections.

For an unclear reason, the regression analysis showed that employed participants were significantly at a higher risk of acquiring parasitic infections than the unemployed. The type of toilet facility used by the participants also posed a significantly high risk of getting infected with intestinal parasites as the odds ratios among patients practicing open defecation and/or using pit latrines were higher than among those who used water closets. Particularly, HIV-positive individuals who do not use a water closet were found to be 53 times more likely to acquire intestinal parasitic infections. This finding agrees with earlier reports in Nigeria [4] and Ethiopia [26].

## 5. Conclusion

The lower infection prevalence suggests that public health interventions put in place may be yielding significant results. Routine diagnosis is recommended to ensure timely and effective treatment and comprehensive care for HIV patients. Moreover, further studies should be conducted among HIV-infected individuals in Ghana to increase public knowledge.

### 5.1. Limitations

The analysis of only one stool sample might lead to some missed parasitological diagnoses. Again, we could not afford molecular techniques which have higher sensitivity rate than the techniques used. Hence, there might have been certain underdiagnoses.

## Figures and Tables

**Table 1:** Demographic and behavioral characteristics of study participants.

Parameter	Frequency	Percentage
Age categories		
≤35	112	33.43
>35	223	66.57

Educational background		
None	252	75.22
Basic	17	5.07
Secondary	64	19.10
Tertiary	2	0.60

Marital status		
Single	167	49.85
Married	168	50.15

Employment status		
Employed	182	54.33
Unemployed	153	45.67

Type of toilet facility		
Open defecation	103	30.75
Pit latrine	215	64.18
Water closet	17	5.07

Source of feeding		
Home prepared	306	91.34
Buy from outside	29	8.66

Source of drinking water		
Sachet water	198	59.10
Borehole/other	137	40.90

Deworming history		
≤3 months	43	12.84
4–6 months	40	11.94
7–11 months	4	1.19
≥12 months	6	1.79
None	242	72.24

Organized deworming exercise		
Yes	20	5.97
No	315	94.03

Data are presented as frequency and percentages.

**Table 2:** Parasites and their stages identified in stool samples using various detection techniques.

Parameter	Total	Wet prep	FECT	Both wet prep and FECT
Parasitic agent				
*A. lumbricoides*	4 (1.19)	3 (0.90)	1 (0.30)	0 (0.00)
*E. histolytica/dispar*	3 (0.90)	3 (0.90)	1 (0.30)	1 (0.30)
*G. lamblia*	10 (2.99)	10 (2.99)	2 (0.60)	2 (0.60)
*T. trichiura*	3 (0.90)	0 (0.00)	3 (0.90)	0 (0.00)
Total	20 (5.97)	16 (4.78)	7 (2.09)	3 (0.90)

Stage of parasitic agent				
Trophozoites	11 (3.28)	11 (3.28)	—	
Egg	5 (1.49)	5 (1.49)	—	
Cyst	7 (2.09)	—	7 (2.09)	

Data are presented as figures with corresponding percentages in parentheses. FECT = formol-ether concentration techniques; Prep = preparation.

**Table 3:** Logistic regression analysis on sociodemographic and behavioral characteristics associated with intestinal parasitic infection among the study participants.

Parameter	IP present	No IP present	*P* value	OR (95% CI)	*P* value
Age					
≤35	6 (1.79)	106 (31.64)	0.8119	1.02 (0.296 to 3.507)	0.977
>35	14 (4.18)	209 (62.39)		Ref	Ref

Education					
None	14 (4.18)	238 (71.04)	**0.0002**	–	–
Basic	1 (0.30)	16 (4.78)		0.94 (0.116 to 7.617)	0.955
Secondary	3 (0.90)	61 (18.21)		1.20 (0.333 to 4.294)	0.784
Tertiary	2 (0.60)	0 (0.00)		Ref	Ref

Marital					
Single	11 (3.28)	156 (46.57)	0.6532	2.09 (0.275 to 15.927)	0.475
Married	9 (2.69)	159 (47.46)		Ref	Ref
Employment					
Employed	7 (2.09)	175 (52.24)	0.1037	6.34 (1.082 to 37.123)	**0.041**
Unemployed	13 (3.88)	140 (41.79)		Ref	Ref

Toilet facility type					
Open defecation	7 (2.09)	96 (28.66)	0.0854	12.17 (0.390 to 379.756)	0.155
Pit latrine	10 (2.99)	205 (61.19)		53.00 (1.512 to 1858.251)	**0.029**
Water closet	3 (0.90)	14 (4.18)		Ref	Ref

Source of feeding					
Home prepared	18 (5.37)	288 (85.97)	0.6876	1.2 (0.2603 to 4.632)	0.34
Buys from outside	2 (0.60)	27 (8.06)		Ref	Ref

Source of drinking water					
Sachet water	10 (2.99)	188 (56.12)	0.483	0.68 (0.2715 to 1.684)	0.5356
Borehole	10 (2.99)	127 (37.91)		Ref	Ref

Deworming history					
≤3 months	1 (0.30)	42 (12.54)	**<0.0001**	20.63 (0.576 to 738.008)	0.097
4–6 months	–	40 (11.94)		–	–
7–11 months	1 (0.30)	3 (0.90)		0.07 (0.002 to 1.983)	**0.117**
≥12 months	1 (0.30)	5 (1.49)		0.23 (0.021 to 2.459)	0.224
None	17 (5.07)	225 (67.16)		Ref	Ref

Deworming exercises witnessed					
Yes	2 (0.60)	18 (5.37)	0.3388	0.54 (0.1302 to 2.535)	0.290
No	18 (5.37)	297 (88.66)		Ref	Ref

Data are presented as frequencies with corresponding percentages in brackets. IP = intestinal parasite, OR = odds ratio, CI = confidence interval, and Ref = reference; *P* value significant at *P* < 0.05.

## Data Availability

Data are obtainable from the corresponding author upon satisfactory request.

## Supplementary Materials

Supplementary Materials The questionnaire used in the study is presented in supplementary Table 1.
