# Supplementary material for: Intestinal Parasitic Infection and Associated Risk Factors among HIV-Infected Patients Seeking Healthcare in a Rural Hospital in Ghana
Source: J Pathog. 2022 Aug 22;2022:5652637. doi: 10.1155/2022/5652637 (PMC9424049; doi:10.1155/2022/5652637)
Supplement: Supplementary Materials — The questionnaire used in the study is presented in supplementary Table 1. [file 5652637.f1.docx]

| Supplementary table 1. Questionnaire used in the study | | |
| --- | --- | --- |
|  | Participant’s identification number | Contact information |
|  | **SECTION B: BIODATA OF RESPONDENTS** |  |
| 1 | How old are you? |  |
| 2 | Formal education | None Basic  Secondary Tertiary |
| 3 | Marital status | Single Married  Divorced Others |
| 4 | Employment of respondents | Unemployed Employed Specify |
| **SECTION C: PERSONAL FACTORS THAT MAY PREDISPOSE RESPONDENTS TO STUDY OUTCOMES** | | |
| 5 | What type of toilet facility do you use? | Pit latrine Water closet  Public toilet Others |
| 6 | Do you share the toilet facility with others? | Yes No |
| 7 | Do you wash your hands after defecation? | Yes No Sometimes |
| 8 | What is your major source of feeding? | Prepare food at home Buy food outside  Others |
| 9 | Do you wash your hands before and after eating? | Yes No |
| 10 | If yes, with what? | Soap and water Water only |
| 11 | What is your main source of drinking water? | Pipe-borne water Borehole  Well River/ Stream Sachet |
| 12 | When was your last deworming date? | ≤3 months 4-6 months 7-11 months  ≥12months Not at all |
| 13 | Has any deworming exercise been organized in the community recently? | Yes No |
